# Quality of anthropometric data measured in children and adolescents with cystic fibrosis: a scoping review

**DOI:** 10.1590/1984-0462/2023/41/2021333

**Published:** 2023-03-03

**Authors:** Fernanda Martins Dias Escaldelai, Luiz Vicente Ribeiro Ferreira da Silva, Lenycia de Cassya Lopes Neri, Denise Pimentel Bergamaschi

**Affiliations:** a Universidade de São Paulo, São Paulo, SP, Brazil

**Keywords:** Adolescent, Body composition, Body mass index, Cystic fibrosis, Child, Adolescente, Composição corporal, Índice de massa corporal, Fibrose cística, Criança

## Abstract

**Objective:**

This study aimed to identify methodological aspects involved in determining anthropometric measurements among studies assessing the nutritional status of individuals with cystic fibrosis (CF).

**Methods:**

A search of the literature was performed on MEDLINE via Pubmed, Embase, and Web of Science databases. The population comprised children and adolescents with CF. Observational studies and clinical trials using anthropometric and body composition measures and indices determined by dual-energy X-ray absorptiometry (DXA) and bioelectrical impedance assessment (BIA) were included. Use of a standardized procedure for data collection was defined when details on the instruments and their calibration were given, the measuring procedures were described, and when it was clear measures had been determined by a trained team, or the use of an anthropometric reference manual was cited. Data extracted were expressed as absolute and relative frequencies.

**Results:**

A total of 32 articles were included, and a total of 233 measures or indices were observed. The most frequently used measures were body mass index (kg/m^2^; 35%), weight (kg; 33%), and height (cm; 33%). Among the 28 studies that used anthropometric measures, 21 (75%) provided a complete or partial description of the measurement instruments used, 3 (11%) reported information on equipment calibration, 10 (36%) indicated the measurement procedures employed by assessors, and 2 (7%) stated a trained team had carried out the measurements.

**Conclusions:**

The poor description of measuring procedures precluded a meaningful evaluation of data quality. Scientific debate on this theme can help raise awareness of the need to ensure quality in collecting and fully presenting data.

## Resumo

**Objetivo:**

Identificar os aspectos metodológicos relacionados à aferição de medidas antropométricas em pesquisas que avaliaram o estado nutricional de indivíduos com fibrose cística (FC).

**Métodos:**

Realizou-se busca nas bases *Medical Literature Analysis and Retrieval System Online* (MEDLINE) via *United States National Library of Medicine* (PubMed), *Embase* e *Web of Science*. A população foi composta de crianças e adolescentes com FC. Foram incluídos estudos observacionais e ensaios clínicos que utilizaram medidas e índices antropométricos ou dados de composição corporal obtidos pela *dual-energy X-ray absorptiometry* (DXA) e bioimpedância elétrica (BIA). Considerou-se o uso de procedimento padronizado na coleta dos dados quando o estudo apresentou informações sobre os instrumentos, a calibração dos equipamentos, os detalhes dos procedimentos de mensuração e a realização de treinamento. Foram apresentadas as frequências absolutas e relativas dos dados extraídos.

**Resultados:**

Trinta e dois artigos foram selecionados, sendo observadas 233 medidas ou índices. Desse total, os mais utilizados foram índice de massa corporal (kg/m^2^; 35%), peso corporal (kg; 33%) e altura (cm; 33%). De 28 estudos que utilizaram medidas antropométricas, 21 (75%) apresentaram detalhes totais ou parciais dos instrumentos utilizados nas aferições, três (11%) apresentaram informações sobre a calibração dos equipamentos, dez (36%) apresentaram a indicação dos procedimentos de mensuração utilizados pelos avaliadores e dois (7%) mencionaram a realização de treinamento da equipe responsável pelas aferições.

**Conclusões:**

Há pouco detalhamento dos procedimentos de mensuração nas publicações, dificultando a avaliação da qualidade dos dados. O debate científico sobre o tema pode contribuir para conscientizar sobre a necessidade de assegurar a qualidade na coleta dos dados e sua apresentação completa nos artigos.

## INTRODUCTION

Cystic fibrosis (CF) is a hereditary recessive genetic disease that affects several organs and systems because of dysfunction of the CFTR (*cystic fibrosis transmembrane conductance regulator*) protein.^1^ In spite of therapeutic improvements in recent years, according to the Brazilian Cystic Fibrosis Registry (*Registro Brasileiro de Fibrose Cística*), the median age at death is 19.4 years.^2^ Malnutrition and recurrent pulmonary exacerbations are recognized strong predictors of CF mortality.^3^

Close monitoring of nutritional status is recommended for CF individuals, through periodical follow-up of anthropometry and body composition parameters.^4^ The goal for pediatric CF individuals is to attain a body mass index for age (BMI/A) above the 50th percentile because this is associated with better pulmonary function.^5^ Weight gain should thus take place through an increase in lean mass as opposed to body fat only. Besides BMI monitoring, body composition evaluation methods such as skinfolds measurement, dual-energy X-ray absorptiometry (DXA), and bioelectrical impedance assessment (BIA) are recommended.^4,6^

The most used method for nutritional assessment, anthropometry, is frequently associated with measurement errors in patients, introduced by instruments, operators, or changes in the subjects’ body composition.^7^ Since the validity of results of any survey depends on the absence of systematic methodological errors,^8^ measurements such as weight and height must be obtained through accurate procedures to be suitably interpreted.^9^

The objective of this study was to identify methodological aspects involved in determining anthropometric measurements among studies assessing the nutritional status of children and adolescents with CF. Preliminary searches of the International Prospective Register of Systematic Reviews (PROSPERO) and JBI Evidence Synthesis revealed no previous reviews on the topic, justifying the conducting of the present scoping review.

## METHOD

The study was carried out based on the recommendations of the Joanna Briggs for scoping reviews (https://jbi.global), and the study protocol was registered with the Open Science Framework (https://osf.io/q2acf/). A systematic search of the literature was performed on MEDLINE via PubMed, Embase, and Web of Science databases. The following research questions were addressed:

1. Which anthropometric and body composition measures are used for evaluating the nutritional status of children and adolescents with CF in clinical practice and in research?
2. Are standardized procedures employed to obtain these anthropometric measurements? and
3. Which reference populations are used to classify the nutritional status of people with CF?

The search criteria were based on the PCC strategy (population, concept, and context). The population comprised children and adolescents with CF of both genders. Concept was defined as anthropometric data, including the use of simple anthropometric measurements:^9^ body weight, body height, waist circumference, and skinfolds; the use of anthropometric indices: weight-for-age, height-for-age, BMI, and BMI/A; and the use of body composition-related measurements or indices obtained by BIA, DXA, or an equation: lean mass, body fat mass, and percentage of fat. Context was defined as CF.

Eligibility criteria were as follows: articles that included children and adolescents with CF, age 6–18 years, even if individuals of other ages were also assessed; involved evaluation of nutritional status or evolution of childhood growth, or the statistical relationship of anthropometric and body composition measurements with clinical outcomes, such as pulmonary function; and used at least one of the anthropo-metric measurements or indices cited in the main concept of the present study.

Exclusion criteria were as follows: studies that included hospitalized subjects, individuals on a lung, pancreas, or liver transplantation waiting list, those who had undergone any type of transplant surgery, pregnant women, and individuals with associated conditions such as celiac disease, Crohn’s disease, or cancer. Experimental studies assessing the pharmacodynamics and pharmacokinetics of drugs or studies with self-referenced body composition or anthropometric measurements were also excluded.

The search strategy was defined by two reviewers (FMDE and DPB) who conducted independent searches. The search included observational studies or clinical trials pursuant to the study objective. To make the scoping review possible, the studies included were restricted to those published between January 2014 and December 2021 and to those articles in English and Portuguese.

**Figure 1.**
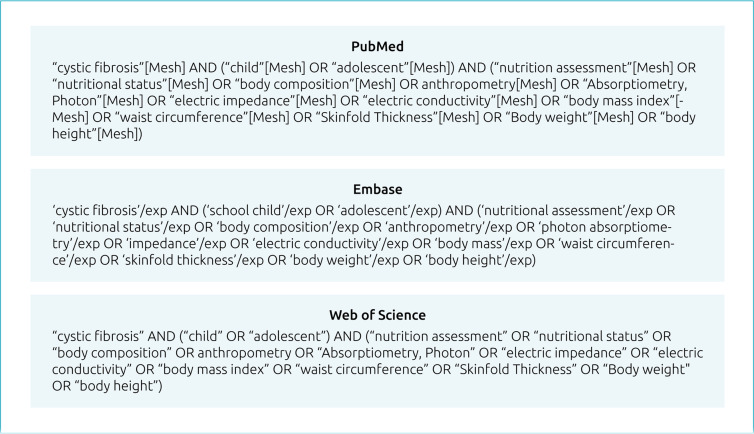
Search terms applied in databases selected.

Specific descriptors for each database were used: MeSH terms (Medical Subject Head) in Pubmed and thesaurus Emtre® in Embase®. Initially, keywords associated with the PCC acronym were searched in Pubmed and then in the other databases; adaptations were made when no corresponding matches were found in the MeSH terms. The terms were linked by Boolean operators AND (restriction) and OR (addition) and constituted search phrases (Figure 1) used on the databases. The use of EndNote online (Clarivate Analytics, Boston, MA, USA) helped manage and organize the studies retrieved, removing duplicates.

An initial selection of the studies was made based on titles and abstracts, independently by two authors (FMDE and DPB). When abstracts did not include age group or a description of anthropometric or body composition measurements, the methods section of the article was consulted. Differences were resolved by consensus.

As part of the selection process, two independent reviewers (FMDE and DPB) applied the Downs and Black checklist.^10^ The original checklist contains 27 questions and was devised and validated to assess the methodological quality of observational studies and clinical trials on the domains of reporting, external validity, internal validity (bias), confounding/selection bias, and power. Because the present study included articles with different designs, only questions 1, 2, 3, 5, 6, 7, 10, 11, 12, 16, and 20 were applied. Differences were resolved by consensus. The maximum possible score for each article was 12 points. Articles scoring 9 or more points were considered eligible. For question 20 [“Were the main outcome measures used accurate (valid and reliable)*?*”],^10^ only the methodological aspects pertaining to the use of anthropometric and body composition data to focus on the abovementioned concept were evaluated.

Data were extracted to characterize the studies and methodological aspects that allowed the use of standardized anthropometric data collection procedures,^9^ namely,

1. Anthropometric and body composition measures and indices used;
2. Source of each measurement – either by direct measurement or extracted from medical record;
3. Details on instruments, such as manufacturer, type, and scale;
4. Calibration of instruments;
5. Details on measuring procedures, such as descriptions of measuring techniques, clothing worn, and presence of accessories;
6. Training given;
7. Use of anthropometric reference manual;
8. Reference curves; and
9. Criteria for grading nutritional status.

The data extracted were recorded by the two independent evaluators (FMDE and DPB) using a chart. Differences were resolved by consensus.

The data obtained were analyzed using the statistical software package Stata version 13 (Stata Corp LP, TX, USA) and expressed qualitatively (descriptive text) or quantitatively (in tables) in the form of absolute and relative frequencies.

## RESULTS

**Figure 2.**
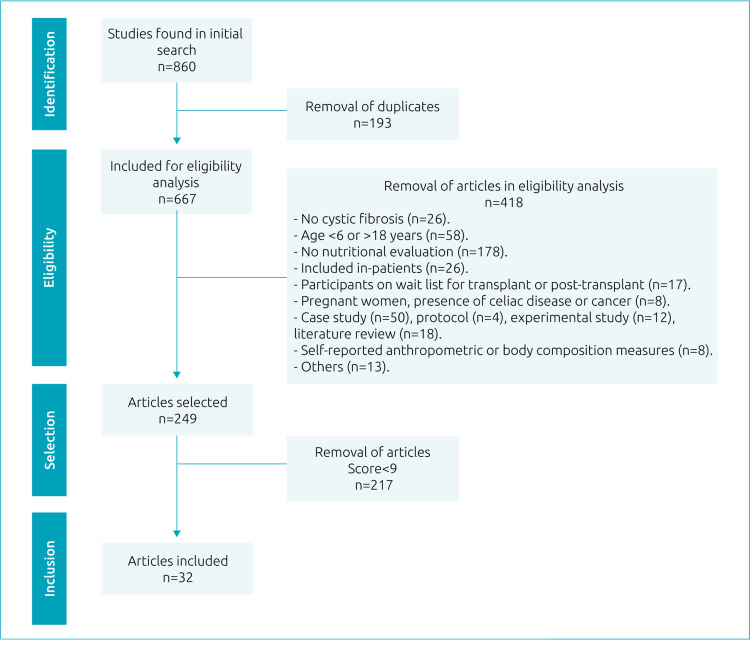
Flow diagram of process of article search, selection, and inclusion.

The initial search resulted in the retrieval of 667 articles, excluding duplicates; 249 (37%) of these were subsequently selected. Of this initial total, 32 (13%) articles were considered eligible and included in this scoping review (Figure 2; Tables 1 and 2).^11-42^

**Table 1.** Characteristics of studies included in scoping review (2014–2018).

Author and year	Study type*	Country	Sample size
Bizzarri et al., 201^51^1	2	Italy	CF and CFRD group: 17. Control group CF, normal GTT: 52
Bruzzese et al., 2018^12^	3	Italy	*Lactobacillus rhamnosus* GG group: 41 Placebo group: 40
Calella et al., 2018^13^	1	The United Kingdom	69
Dentini et al., 2017^14^	1	Brazil	Group A (BCC): 50. Group B (non-BCC): 134
Doulgeraki et al., 2017^15^	1	Greece	HMI: 19. Without HMI: 82
El Attar et al., 2017^16^	1	Egypt	50
Engelen and Deutz, 2014^17^	1	The United States	CF group: 15. Control group: 17
González-Jiménez et al., 2017^18^	1	Spain	Pancreatic sufficiency: 371. Exocrine pancreatic insufficiency: 80
Goss et al., 2015^19^	1	The United States and United Kingdom	13,777; 3,968
Goss et al., 2018^20^	2	Canada and the United States	5,149; 37,772
Groleau et al., 2014^21^	3	The United States	LXS group: 27. Placebo group: 36
Haack and Garbi-Novaes, 2014^22^	1	Brazil	Without deficit: 34. With deficit: 13
Hauschild et al., 2016^23^	1	Brazil	CF group: 46. Group without CF: 24
Hauschild et al., 2018^24^	2	Brazil	38
Hortencio et al., 2015^25^	2	Brazil	52
Isa et al., 2016^26^	1	Bahrain	47
Kelly et al., 2016^27^	1	The United States	CF group: 97. Control group: 199
Ledder et al., 2015^28^	2	Australia	59
Okuro et al., 2017^29^	1	Brazil	CF group: 55. Control group: 185
Ong et al., 2017^30^	2	The United States	1,375
Papalexopoulou et al.,2018 ^31^	1	The United Kingdom	CF group in study 1: 18. Study 2: 29
Sands et al., 2015^32^	1	Poland	89
Sheikh et al., 2014^33^	1	The United States	CF group: 208. Control group: 390
Welsh et al., 2014^34^	2	Australia	98
Woestenenk et al., 2017^35^	1	Holland	474

*Type of study: 1: cross-sectional; 2: longitudinal: 3: clinical trial. BCC: *Burkholderia cepacia complex*; CF: cystic fibrosis; CFRD: cystic fibrosis-related diabetes; HMI: history of meconium ileus; LXS: LYM-X-SORB™; GTT: glucose tolerance test.

**Table 2.** Characteristics of studies included in scoping review (2019–2021).

Author and year of publication	Study type*	Country	Sample size
Barbosa et al., 2020^36^	1	Brazil	31
Campos et al., 2020^37^	1	Brazil	CF group: 30. Control group (healthy individuals): 25
Hak et al., 2019^38^	2	Holland	57
Owen et al., 2021^39^	1	The United Kingdom	37
Phong et al., 2020^40^	1	The United States	49
Poulimeneas et al., 2020^41^	1	Greece	76
Sainath et al., 2019^42^	2	The United States, Canada, and Italy	22

*Type of study: 1: cross-sectional; 2: longitudinal. CF: cystic fibrosis.

Regarding study type, 22 (69%) were cross-sectional, 8 (25%) were longitudinal, and 2 (6%) were clinical trials. Studies were mostly carried out in Europe (34%), South America (25%), and North America (22%). Sample size ranged from 15 to 42,921: 24 (75%) studies involved fewer than 100 subjects with CF, 5 (16%) had 101–500 subjects, and 3 (9%) included more than 500 subjects (Tables 1 and 2).^11-42^

The articles employed, on average, seven measures or indices (median=6.5; quartile 1=4; quartile 3=10; values=1–19). Overall, a total of 233 measures or indices were used, predominantly derived from anthropometry (n=165; 71%) (Table 3).^11-42^ For anthropometric data, a similar proportion of measures (52%) and indices (48%) were employed. The most frequently used anthropometric measures were body weight (n=28; 33%), height (n=28; 33%), and BMI (n=28; 35%). For body composition, DXA was the most frequently used measuring technique (n=55; 81%); lean mass was determined by DXA in 25% (n=14) of measurements.

**Table 3.** Distribution of anthropometric and body composition measures and indices, by measuring method cited in articles.

Method	n (%)	n (%)
Anthropometry	165 (71)	
Measurements		
Weight		28 (33)
Height		28 (33)
Others		29 (34)
Total		85 (100)
Indices or equations		
Body mass index		28 (35)
Height for age		9 (11)
Body mass index for age		7 (9)
Target height		6 (8)
Weight for age		5 (6)
Upper arm muscle area		4 (5)
Upper arm fat area		3 (4)
Others (chest index, height velocity, hip to shoulder width index, % ideal body weight, puberty status, ratio of leg or trunk length to height, the sum of the three skinfold thickness, weight for height)		18 (22)
Total		80 (100)
Body composition	6,8 (29)	
Dual-energy X-ray absorptiometry		
Lean mass measurements		14 (25)
Lean mass index		10 (18)
Fat mass measurements		10 (18)
Fat mass index		10 (18)
Bone mineral (density and content)		11 (20)
Total		55 (100)
Bioimpedance		
Fat free mass; fat mass		7 (54)
Resistance/height; reactance/height; resistance index		3 (23)
Hydration status; phase angle		3 (23)
Total		13 (100)
TOTAL	233 (100)	

Of the total anthropometric measurements reported (n=85), 60 (71%) comprised primary data collected during study execution, 17 (20%) were secondary data obtained from medical records, and 6 (7%) involved data extracted from patient Registries, while for 2 (2%) studies, the first measurement was performed directly and the remainder was obtained from medical records.

Of the total studies (n=28) which used anthropometric measures, 21 (75%) reported full or partial details on the measuring instruments applied, 3 (11%) provided information on instrument calibration, 10 (36%) detailed the measuring procedures applied by evaluators, and 2 (7%) reported giving training to the team that took the measurements. The anthropometric measures employed by the studies, together with methods applied, are listed in Table 4.

**Table 4.** Description of anthropometric measures cited in articles.

Measurements	Total	Details of instruments		Use of calibrated instruments		Details of measuring procedures		Training given	
	n	n	%	n	%	n	%	n	%
Abdominal skinfold thickness	1	1	100	0	-	1	100	1	100
Birth weight	2	1	50	0	-	0	-	0	-
Height	28	22	79	0	-	10	36	2	7
Hip width	1	1	100	0	-	1	100	1	100
Leg length	1	0	-	0	-	1	100	1	100
Mid-upper arm circumference	6	4	67	0	-	3	50	1	17
Parental height	7	3	43	0	-	0	-	0	-
Sagittal chest depth	1	1	100	0	-	1	100	1	100
Shoulder width	1	1	100	0	-	1	100	1	100
Subscapular skinfold thickness	2	2	100	0	-	1	50	1	50
Transverse chest width	1	1	100	0	-	1	100	1	100
Triceps skinfold thickness	5	4	80	0	-	2	40	1	20
Trunk length	1	0	-	0	-	1	100	1	100
Weight	28	22	79	3	11	9	32	2	7
**Total**	**85**	**63**		**3**		**32**		**14**	

Three (4%) articles cited the use of reference manuals^7,9^ to define the measuring procedures.

The most commonly cited reference populations were those of the Centers for Disease Control and Prevention (CDC)^14,19-21,27,29,31,33,40-42^ and of the World Health Organization (WHO);^18,22-26,37^ however, references were also made to specific populations such as Italian,^11^ Greek,^15^ Egyptian,^16^ Dutch,^35,38^ and British.^39^ The cutoff points adopted by the authors^17,18,22,24,39,42^ were mainly derived from consensuses or specific recommendations for CF subjects.^5,6,43,44^

## DISCUSSION

This review identified anthropometry as the most widely used tool for nutritional assessment of individuals with CF, where BMI (35%), body weight (33%), and height (33%) were the most commonly reported measures. Few articles described the methods used to obtain measurements, hampering the assessment of these aspects. Ascertaining the quality of the technical procedures adopted in the studies and in clinical practice is the key to minimize misclassifications of nutritional status, which may impact clinical and nutritional approaches in treatment planning.

Methodological studies with a focus on assessing the quality of anthropometric measures in CF are scarce.^45^ This critical review of the leading articles concerning nutritional assessment of CF individuals revealed that, although the instruments used for most of the anthropometric measurements were reported, very few studies described calibration procedures. This finding implies a significant risk of systematic errors. In addition, few studies described measuring procedures in detail, compromising reliability and affecting the potential for comparisons between reference values and other values of interest. In addition, few measurements were taken by trained investigators, and the use of standardized procedures by others studies remained merely implicit.

The “Consensus Report on Nutrition for Pediatric Patients” with CF^43^ states that each specialized center should maintain calibrated anthropometric instruments and measurement protocols and also perform team training, in order to ensure reliable measurements. Since many studies included in this review used data taken from medical records, these recommendations seem to be critical to avoid inaccurate conclusions. Studies carried out using medical record-based data may not be reliable due to failures in recording of the data, different approaches used to obtain measurements, or missing data from the medical records.^46^ The same criticism may be applied to studies based on patient registries, since these may include the additional bias of methodological heterogeneity in measurements collected by different institutions. This possible source of error can be reduced by adopting standard criteria for datum measurement^47^ and quality control during the collection of data. Professional capacity building and the standardization of techniques may also improve the precision and accuracy of measurements.^48,49^

According to Fleiss,^50^ the importance of error-free measurements lies in the fact that statistics are incapable of correcting an unreliable datum. Unreliable data may lessen the strength of correlation between variables, result in incorrect decisions for tests of hypotheses, and reduce the power of statistical tests, leading to the need to increase the sample size.

Since CF is a fairly rare disease, studies involving patients with CF include relatively small samples, generally fewer than 100 patients. The current review identified studies that included only 15^17^ and 17 subjects with CF.^11^ As the power of the statistical test is intrinsically related to sample size, a difference between groups may not be detected when the sample size is too small. The decision to include all patients may mitigate precision issues, but if the measurements are not standardized, other methodological errors may remain and jeopardize the internal validity of the results.^8^ Measurement errors in the nutritional evaluation of individuals with CF may have significant implications for the patient’s overall status and impact treatment decisions.

The finding that anthropometry was the most frequent measure adopted in the nutritional studies was expected, given that they have validated psychometric properties, are readily collected, and are of low cost. The ability of BMI to evaluate body composition is still a matter of debate,^51,52^ and DXA has emerged as the preferred approach to investigate body composition in the case of individuals with CF.^4^ However, its high cost has been a constraint to broader application of the technique.

Since people with CF have the potential to grow normally, there is no specific growth curve for this group. The usual reference values for the general population, such as the CDC and WHO curves, were the most frequently adopted in the studies.

The studies describing body fat and lean mass used equations derived from anthropometric measurements, resistance and reactance measurements of BIA, or measurements provided by DXA. These data may have intrinsic limitations, since the equations may have been constructed for other populations or be specific for the manufacturers of the DXA apparatus.

Limitations of the present study included constraints on the search period and language of publication. The defining of the score for considering a study eligible was arbitrary and may have led to the exclusion of some important articles. However, all restrictions served to reduce the number of articles and make the review possible. No complementary searches of other sources, such as gray literature, were carried out, given the articles obtained included different anthropometric and body composition methods generally recommended.

The findings of this review highlight the key role played by hospitals and health services as a source of data in CF. Nutritional assessment is important to guide decisions on patient treatment. Scientific debate on this theme can help raise awareness of the need to ensure quality in collecting and presenting complete data, allowing readers to analyze the internal validity of the studies.

In studies evaluating nutritional status of children and adolescents with CF, few details were given on the measurement procedures used to obtain the commonly used anthropometric measures, hindering a more far-reaching analysis of the nutritional picture in CF and aggregation of data.
